# Sir James Mackenzie on Heart Affections

**Published:** 1916-09-02

**Authors:** 


### A MEDICAL CLASSIC.

### Sir James Mackenzie on Heart Affections.

Since the first appearance of Mitchell Bruce's "Principles of Treatment" there has been no book on any branch of medicine published in England so well calculated to make physicians think as this new one by Sir James Mackenzie.* Epoch-making as his individual contributions and those of his school to scientific medicine—chiefly, but by no means entirely, in cardiology—have already been, the latest development of his industry will perhaps prove even more notable; for here he lays down some of the principles which must guide future advance, and he makes all practitioners who are in the least keen on their profession for its own sake his disciples and colleagues. The general outlines of the "new cardiology" are limned with a sure hand and a deft touch; and there are few men indeed in the medical profession, whether consultants, heart specialists, or general practitioners, who can read this wonderfully illuminating sketch of main principles without largely increasing both their stock of knowledge and their grasp of the means by which recent advances have been made and further progress is reasonably to be expected.

### "Mackenzie of Burnley."

The book is cast in the form of post-graduate lectures, which, owing to the war, have not been delivered. It assumes a working knowledge of physiology and pathology, and to some degree is unsuited therefore for the elementary student. The original design as lectures entails a more dogmatic tone and permits a more personal note than a stereotyped textbook requires; references to literature, for instance, are quite absent. There is an element of reminiscence, too, when the author deals with many of his own numerous researches and discoveries, which is often very interesting. Indeed, the light which the book throws on "Mackenzie of Burnley," as until eight or nine years ago he was usually known, shows the medical world something of a very striking figure. A general practitioner in that town for nearly thirty years, Mackenzie seems to have pursued research unceasingly: not separate from, but along with and part of his private practice. Gradually his contributions to science attracted more and more attention, until by the time he came to London to appointments at the London and the Mount Vernon Hospitals, his name and reputation were in the mouths of everyone interested in the study of heart diseases. With what tireless persistency and boundless energy he pursued his researches, this book gives us some idea. To follow the career of a patient with a diseased heart for fifteen, twenty, or more years, and then to verify his diagnosis and his conclusions by autopsy, seems to have been nothing uncommon; clearly one of his gifts in practice was that of retaining the unlimited confidence of his patients.

From the thesis that in hospitals, clinical laboratories, and specialists' consulting-rooms only the later stages of disease are available for study, the author deduces that the future of research lies with the general practitioner. We cannot follow in full detail the arguments with which he supports this conclusion, fascinating and ingenious though they are;* but we cannot forbear the suggestion that Sir James has been arguing unconsciously from the particular to the general. Because he conducted, as a general practitioner, researches of unequalled value, it does not follow that all research will best be thus conducted; his own genius is so unique that the generalisation is doubly unsafe. Further, he will surely allow that the most brilliant of his disciples, who have already in some cases achieved results of real importance and value, have not been general practitioners but specialists. It may be admitted, however, that more research might well be attempted by those in general practice, and that in certain chronic diseases the practitioner's share in future research will have to be greater than in the past if the best results are to be attained. Sir James's discussion of this question deserves the most careful attention of every medical man.

### The New Cardiology.

After this opening disquisition upon research, the author proceeds to an exposition of his views upon the principles of cardiac disease. These he sets forth in simple, lucid teaching, arranged with the most admirable precision and the most unanswerable logic. The fact that in very many respects the teaching completely upsets views which the day before yesterday, as it were, were implicitly believed and acted upon by the entire profession of medicine is one that Sir James does not fail to dwell upon. Beyond all doubt he has demolished finally and for ever many venerated doctrines, though a good many members of the profession brought up therein have not yet forsaken them. But now and then he is a little hard upon some of his predecessors. Broadbent, for instance, was fully seized with the importance of the cardiac

* *Principles of Diagnosis and Treatment in Heart Affections.* By Sir James Mackenzie, M.D., F.R.S. London: Henry Frowde and Hodder and Stoughton. 1916. Pp. 264. Price 7s. 6d. net.

* Elsewhere in this issue another view of the future of research will be found set forth (see p. 505).

muscle as the main factor in the prognosis of heart lesions; where he failed was in his inability to estimate its quality, not for want of attempts to do so, but for want of precisely those standards and tests which Sir James Mackenzie's work has supplied.

Among the many excellencies of this book, one cannot forbear to mention the castigation administered to the Nauheim and similar "treatments" for heart disease which are run on a basis of commercialised quackery by German doctors and their English imitators. Some plain-speaking about this subject has long been needed, and from an authority of the author's standing it comes with peculiar force and with a good chance of acceptance. Possibly after this war the average English patient will have ceased to worship any form of pretentious treatment merely because it happens to be German. Indeed, the suppression of "eyewash" of all kinds, especially that connected with blood pressure, the clearing away of shams and pretences in favour of truth and scientific accuracy, these are aims which are constantly inculcated throughout the book. There is not a page of it which any reader can afford to skip; it is a veritable classic, both in matter and in manner. It reveals powers of thought and of making other people think which would establish their possessor as a man of genius even if his accomplished works had not already thus revealed him. The medical profession and the State owe this man a debt which is not sufficiently paid by the recognition he has hitherto received. It is true that Universities have conferred honorary degrees upon him; but as far as the State is concerned he has obtained exactly the same honour as the three blameless but quite undistinguished doctors whose title to fame is that they have been the private practitioners in attendance on the present Prime Minister and his two predecessors in that office. Yet he certainly stands head and shoulders above every other practitioner of the day.

